# Uniform 3D meshes to establish normative facial averages of healthy infants during the first year of life

**DOI:** 10.1371/journal.pone.0217267

**Published:** 2019-05-20

**Authors:** Sander Brons, Jene W. Meulstee, Rania M. Nada, Mette A. R. Kuijpers, Ewald M. Bronkhorst, Stefaan J. Bergé, Thomas J. J. Maal, Anne Marie Kuijpers-Jagtman

**Affiliations:** 1 Department of Dentistry, Section of Orthodontics and Craniofacial Biology, Radboud University Medical Centre, Nijmegen, Netherlands; 2 Department of Oral and Maxillofacial Surgery, Radboud University Medical Centre, Nijmegen, Netherlands; 3 Faculty of Dentistry, Kuwait University, Jabriya Kuwait City, Kuwait; 4 Department of Dentistry, Section of Preventive and Curative Dentistry, Radboud University Medical Centre, Nijmegen, Netherlands; Navodaya Dental College and Hospital, INDIA

## Abstract

Three-dimensional (3D) surface imaging systems are replacing direct anthropometry as the preferred method for capturing facial soft-tissues. Aims of this study were: (1) to develop normative average 3D faces of healthy infants aged 3, 6, 9, and 12 months and (2) to describe normative average 3D facial growth data in infants aged 3 to 12 months. Three-dimensional images of 50 healthy children were acquired at 3, 6, 9, and 12 months of age using the 3dMDcranial system. Four average faces with uniform meshes (3, 6, 9, and 12 months) were developed and registered based on the children’s reference frames. Distance maps of growth of the total facial surface and of the nose, upper lip, chin, forehead and cheeks for the intervals 3 to 6 months, 6 to 9 months, and 9 to 12 months of age were calculated. Mean growth of the total facial surface was 3.9 mm (standard deviation [SD] 1.2 mm), 3.5 mm (SD 0.9 mm), and 1.6 mm (SD 0.7 mm) at 3 to 6 months, 6 to 9 months, and 9 to 12 months, respectively. Regarding the selected regions of the face, the mean growth of the nose and upper lip were the largest (3.7 mm and 3.6 mm, respectively) between 6 and 9 months of age. The mean growth of the forehead, cheeks and chin were the largest (5.4 mm, 3.2, and 4.7 mm, respectively) between 3 and 6 months of age. For all facial regions, growth clearly diminished from 9 to 12 months of age. Normative data on the growth of the full face, nose, upper lip, chin, forehead and cheeks are presented. Such data can be used in future studies to identify the effectiveness of treatment of orofacial deformities such as orofacial clefts during the first year of life.

## Introduction

Since the last century, X-ray cephalometric measurements have provided useful data for assessment of deviation of individual morphology from the normal range as well as for studying morphological growth changes of the head and face in response to surgical and orthodontic treatment. The measurements on these two-dimensional cephalograms were primarily profile-oriented and revealed both anteroposterior and vertical relationships of the dentofacial complex. Since the introduction of X-ray cephalometry more than 80 years ago, hundreds of cephalometric studies, including both operated and unoperated cleft individuals, have suggested that deviations from normal facial development are either directly caused by the primary anomaly, and/or by surgical interventions and the subsequent disturbed and compensatory growth of the facial bones [1–8]. A drawback of conventional cephalometric analysis is that it is limited to a lateral two-dimensional projection using simplistic cephalometric analyses for a complex three-dimensional (3D) structure as is the face. Another limitation of conventional cephalometrics is the use of ionizing radiation. Even in infants with orofacial clefts in which radiographic examinations are justifiably obtained to generate diagnostic yield, there is a need for optimization of radiation exposures [9]. Furthermore, since it is unethical to use X-ray imaging in unaffected healthy controls, no normative database on facial growth in babies and young children exists. In 2009, FaceBase, an online collaborative consortium to generate data in support of advancing research into craniofacial development and malformation, was launched [10]. To date, the only normative database on craniofacial growth of healthy newborns and infants is the one of Farkas who used direct anthropometry to describe linear and angular measurements of the head, thereby simplifying the complex 3D nature of craniofacial morphology [11].

However, in recent years medical imaging has moved from two-dimensional representations to more sophisticated 3D techniques [12]. Non-contact 3D surface imaging systems are on their way to replace direct anthropometry as the preferred method for capturing quantitative information about facial soft tissues [13, 14]. The safety, speed and ability of data acquisition are particularly helpful when working with young children, for whom quantification of facial features can be challenging [11, 15]. These systems offer several advantages: non-ionizing radiation, quick image capture and the ability to archive images for subsequent analyses [16]. This potential allows for the collection of normative data of 3D facial growth in healthy control subjects. Such data are important for comparative studies of growth patterns and treatment outcomes in infants with congenital craniofacial malformations, such as orofacial clefts.

The aims of this study were: (1) to develop normative average 3D faces of healthy infants aged 3, 6, 9, and 12 months, and (2) to describe normative average 3D facial growth data in infants aged 3 to 12 months.

## Materials and methods

### Ethical approval and informed consent

The study protocol was approved by the medical ethical commission of the Radboud University Medical Centre (CMO 2007/163). All subjects’ parents provided written informed consent prior to their inclusion in the study. All experiments were performed following the basic tenets in the Helsinki declaration of 1964.

### Subjects

Fifty healthy newborns were enrolled in the study. The subjects were recruited before the age of 3 months between April 2007 and September 2010 at the Maternity Clinic of the Radboud University Medical Centre, Nijmegen, and the Regional Health Services (GGD Gelderland-Zuid). Th inclusion criteria were: (1) healthy, (2) born at term (38+ weeks), and (3) both parents Caucasian. Exclusion criteria were: (1) occurrence of orofacial clefts in first, second or third-degree relatives.

### 3D image acquisition

Acquisition of 3D images was done with the 3dMDcranial System (3dMD Ltd., Atlanta, GA, USA) within a period of +/-7 days around the ages of 3, 6, 9, and 12 months. The 3dMDcranial System was calibrated daily and the set up was permanently located in a room with no windows, with consistent ambient lighting. 3D facial images were constructed from the 2D images acquired by 15 digital cameras using the 3dMDpatient 4.0 software. The image capture duration was 1.5 milliseconds. On each occasion, approximately four images were obtained within 10 minutes, depending on the subject’s cooperation. A quick visual assessment of image quality was conducted immediately after acquisition by the photographer, while completeness of 3D image data and neutral facial expression assessments was performed using the 3dMDpatient V4.0 software.

### Selection of eligible 3D images

High-quality 3D images of the face at rest were selected by one observer (S.Br.). The inclusion criteria were: (1) a neutral facial expression with the eyes open, (2) facial orientation in the natural head position, (3) correct 3D image construction, (4) no data holes in the facial region medial to the ears, caudal to the hairline and cranial to the menton, and (5) no presence of a cap or pacifier. Reliability of the selection procedure was assessed by independent selection of a sample of images by two observers (S.Br. and R.N.), as described in a previous publication [17].

### 3D image processing

The selected 3D images were first exported from the 3dMDpatient 4.0 software as wave front object (.obj) files with texture. Following this, the 3D images were imported into Maxilim version 2.3.0.3 (Nobel-Biocare, Mechelen, Belgium). The children’s reference frame described by Brons et al. was used to align all 3D images in the correct position and orientation [18].

Using the Meshmixer software (Autodesk), remeshing of the 3D images was performed to obtain a uniform mesh pattern with polygon edges of 1.5 mm. MATLAB (MathWorks, MA, USA) was then used to automatically annotate the left and right pupils, the pronasale, and the left and right exostomion on the aligned 3D images. The landmarks were indicated on the 2D texture files automatically with a cascaded convolutional network described by Zhang et al. and transferred to the 3D images [19].

In the fifth step, the 3D images were cropped by selecting only the face of the patient. A general face template was scaled to every individual 3D image by a Procrustes transformation based on the annotated landmarks. After the template was scaled to the individual subjects, the outer boundary of the scaled template was used to crop the 3D images and to remove excess data such as hair, ears, and the neck.

**Fig 1:**
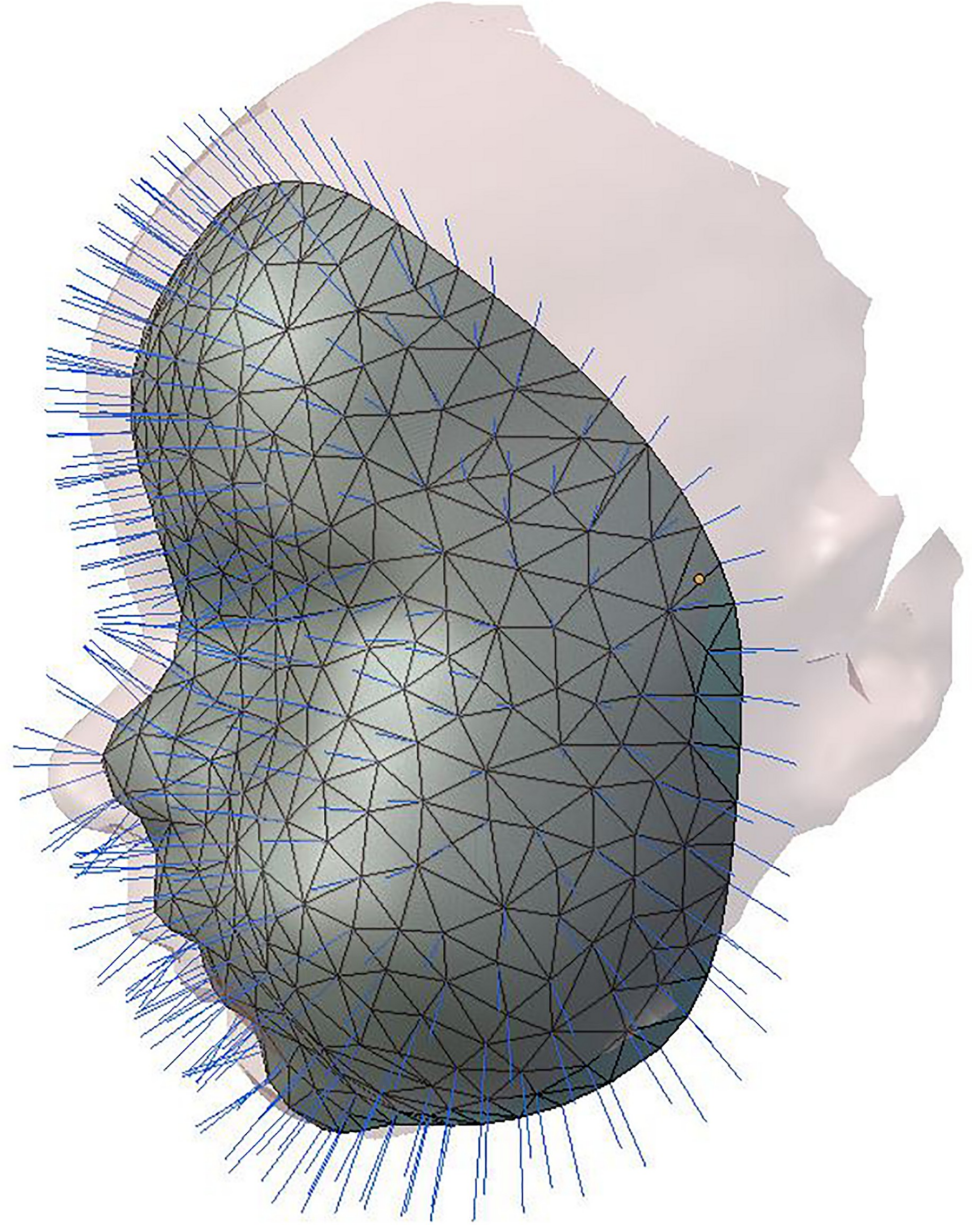
Resampling of 3D Mesh Data to a uniform mesh pattern by casting of rays from the surface of a general face template (Gray) to the surface of the individual 3d image (Purple).

The Coherent Point Drift algorithm was then used for non-rigid deformation of the general face template to the mesh of the 3D images [20]. After this non-rigid transformation of the general face template was done, a resampling by a ray casting algorithm was performed to create a uniform mesh pattern for all subjects with the same number of vertices (Fig 1) [21]. From these uniformly resampled 3D images, gender-neutral average faces were created for ages 3, 6, 9, and 12 months.

**Fig 2:**
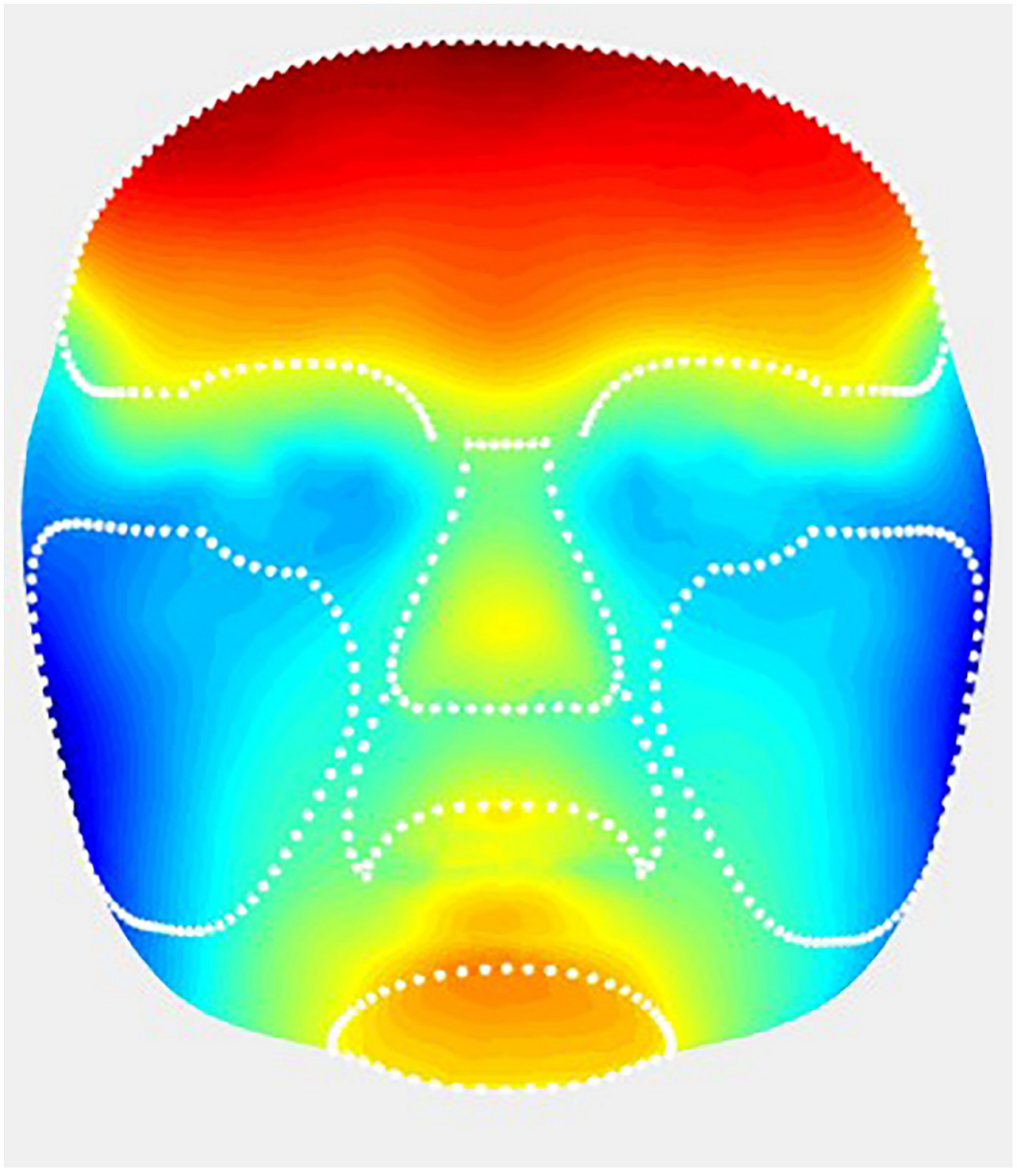
Selected regions for evaluation of facial growth: Nose, upper lip, chin, forehead, and right and left cheeks.

On the general face template (step 5), the regions of the forehead, nose, cheeks, upper lip and chin were selected once, manually (Fig 2) [22]. The selected regions were directly transferred to the average faces for the ages of 3, 6, 9, and 12 months.

Finally, the four average faces (3, 6, 9, and 12 months) were superimposed based on the children’s reference frames to calculate distance maps of the growth of the 3D total facial surface and of the regions of the nose, upper lip, chin, forehead and cheeks for the intervals 3 to 6 months, 6 to 9 months, and 9 to 12 months of age.

### Statistical analyses

Color-distance maps are presented for visual assessment of growth. The growth of the face and regions (nose, upper lip, chin, forehead and cheeks) are presented as the mean growth, standard deviation of the mean, p5 and p95. Density plots were calculated to estimate the distribution of growth velocity of the total facial surface and the nose, upper lip, chin, forehead, and cheeks.

## Results

### Image selection

In total, 615 3D images of controls were available for the selection process. After the selection process, 372 images were excluded due to exclusion criteria. From the remaining 243 3D images, 136 3D images were excluded due to being double images of the same patient. A total of 107 single 3D images were included for development of average faces at ages 3, 6, 9 and 12 months. The average faces at the various time points months consisted of 32 3D images for age 3 months, 20 3D images for age 6 months, 31 3D images for age 9 months, and 24 3D images for age 12 months (Table 1).

**Table 1:** Selection process of eligible 3D images of healthy control subjects.

Age	3 months	6 months	9 months	12 months	Total
Children in database (n = 50)	44	42	47	44	
3D images in database	165	144	144	162	615
Excluded images due to exclusion criteria	85	87	81	119	372
Exclusion of double images	48	37	32	19	136
Included 3D images	32	20	31	24	107

### Average faces and visual assessment of facial growth

**Fig 3:**
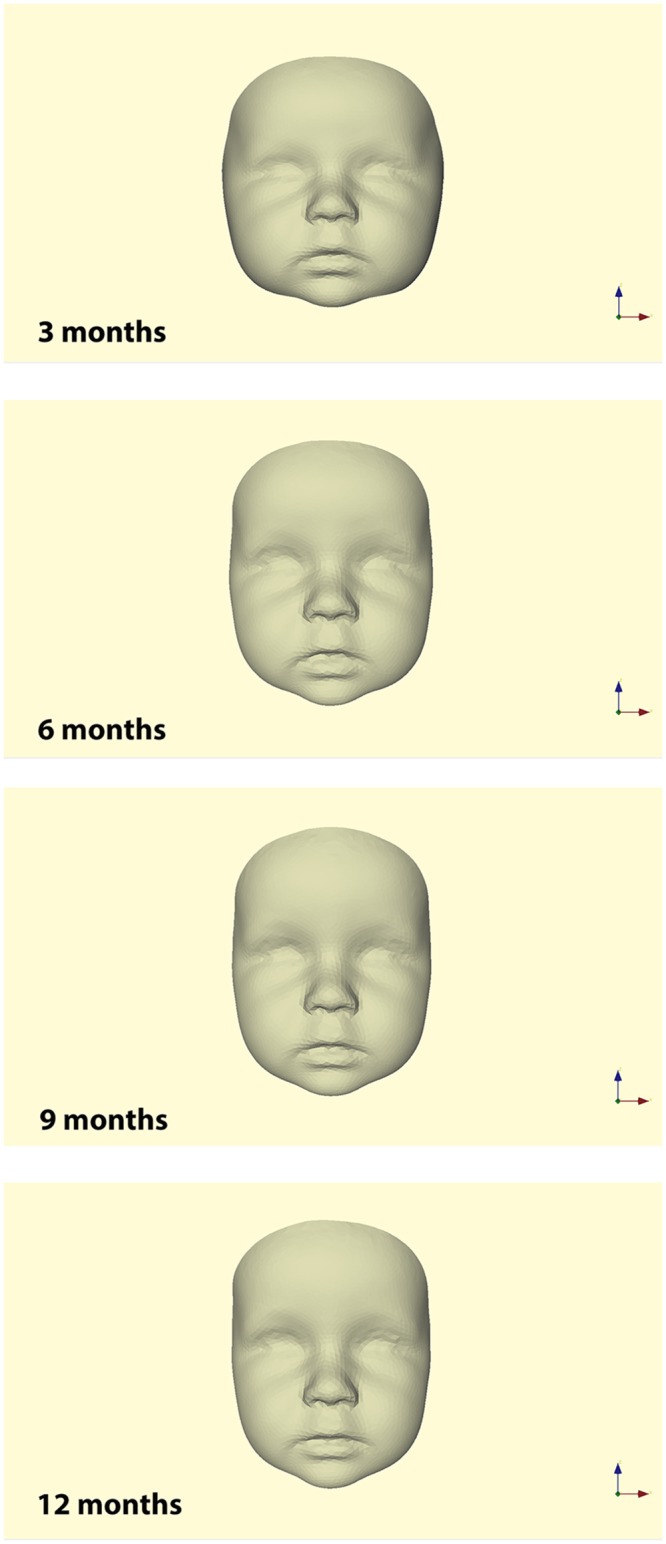
Average faces for healthy controls at 3, 6, 9, and 12 months of age.

**Fig 4:**
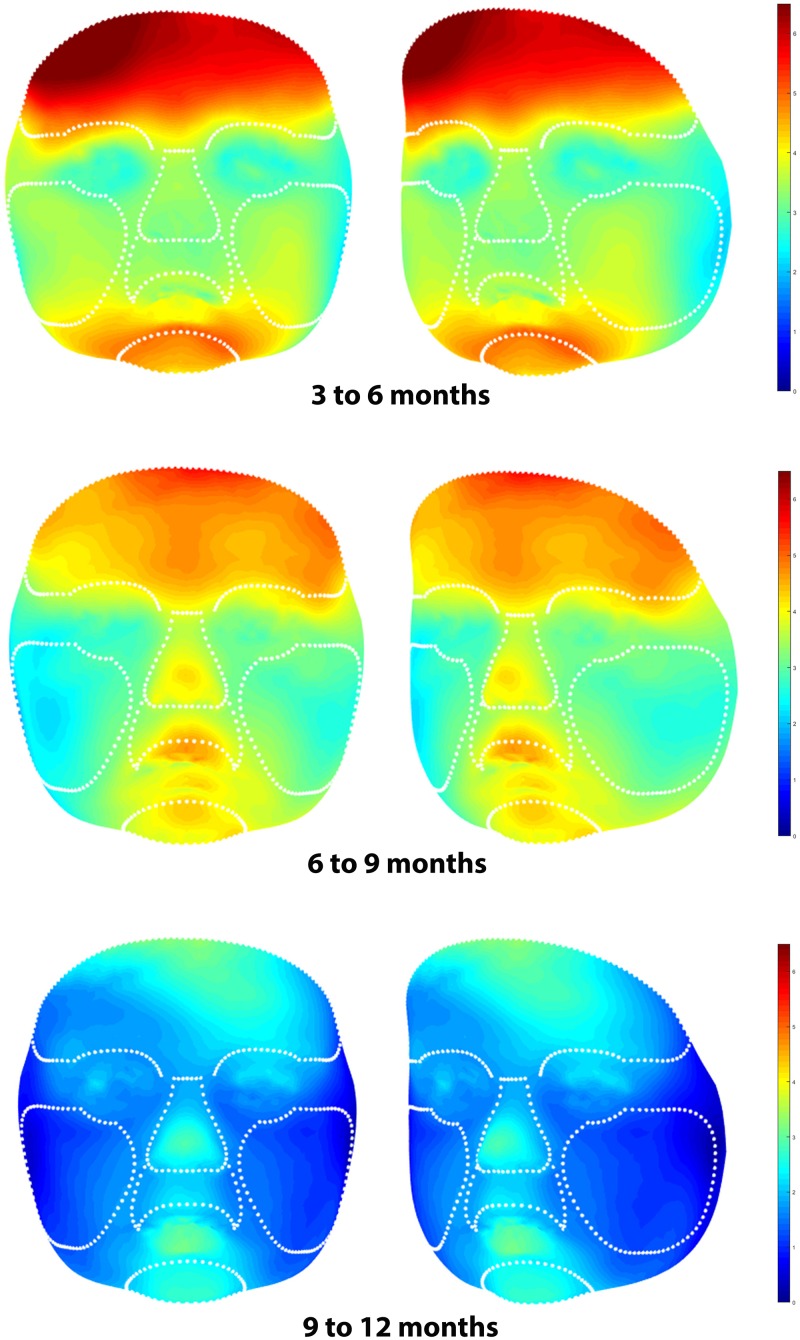
Distance maps of superimposed average faces at 3 and 6 months, 6 and 9 months, and 9 and 12 months of age showing facial growth (Color Scale Blue–Red = 0–6.5 mm).

Average faces for healthy controls at ages 3, 6, 9 and 12 months are presented in Fig 3. Distance maps of the superimposed average faces of 3 and 6 months, 6 and 9 months, and 9 and 12 months of age are presented in Fig 4.

At interval 3 to 6 months of age, the least growth occurred in the region of the eyes and the lateral parts of the cheeks. Growth increased towards the forehead and mandible with the most growth of 5.0 to 6.0 mm occurring towards the forehead and chin.

At interval 6 to 9 months of age, the least growth occurred in the lateral region of the cheeks. Growth increased towards the tip of the nose, lips and chin with the most growth of 5.0 mm occurring at the forehead.

At interval 9 to 12 months of age, growth was less than during the earlier time intervals and more evenly distributed over the face. The most growth of 3.5 mm occurred at the tip of the nose, lower lip, chin, and forehead.

### Growth of the face and facial regions

Mean growth, standard deviation, p5 and p95 of the total facial surface and facial regions are presented in Table 2. Mean growths of the total facial surface were 3.9 mm, 3.5 mm, and 1.6 mm at intervals 3 to 6 months, 6 to 9 months, and 9 to 12 months, respectively.

**Table 2:** Growth of the full face and selected regions of the nose, upper lip, chin, forehead, and cheeks.

	Age period (months)	Mean (mm)	Std. (mm)	p5 (mm)	p95 (mm)
Full face	3–6	3.9	1.2	2.5	6.2
	6–9	3.5	0.9	2.2	4.8
	9–12	1.6	0.7	0.5	2.8
Nose	3–6	3.3	0.1	3.2	3.6
	6–9	3.7	0.3	3.3	4.2
	9–12	2.2	0.3	1.8	2.8
Upper lip	3–6	3.3	0.9	3.1	3.7
	6–9	3.6	1.1	3.3	4.4
	9–12	1.9	0.4	1.5	2.3
Chin	3–6	4.7	0.2	4.2	5.0
	6–9	4.0	0.2	3.6	4.3
	9–12	2.4	0.2	2.1	2.7
Forehead	3–6	5.4	1.0	3.7	6.9
	6–9	4.5	0.4	3.8	5.1
	9–12	2.1	0.5	1.4	3.0
Cheeks	3–6	3.2	0.5	2.4	3.9
	6–9	2.7	0.4	2.1	3.2
	9–12	1.0	0.3	0.4	1.6

Regarding the selected regions of the face, the mean growths of the nose and upper lip were the largest (3.7 mm and 3.6 mm, respectively) between 6 and 9 months of age. Additionally, the mean growths of the forehead, cheeks, and chin were the largest (5.4 mm, 3.2 mm and 4.7 mm, respectively) between 3 and 6 months of age. For all facial regions, growth clearly diminished from 9 to 12 months of age.

**Fig 5:**
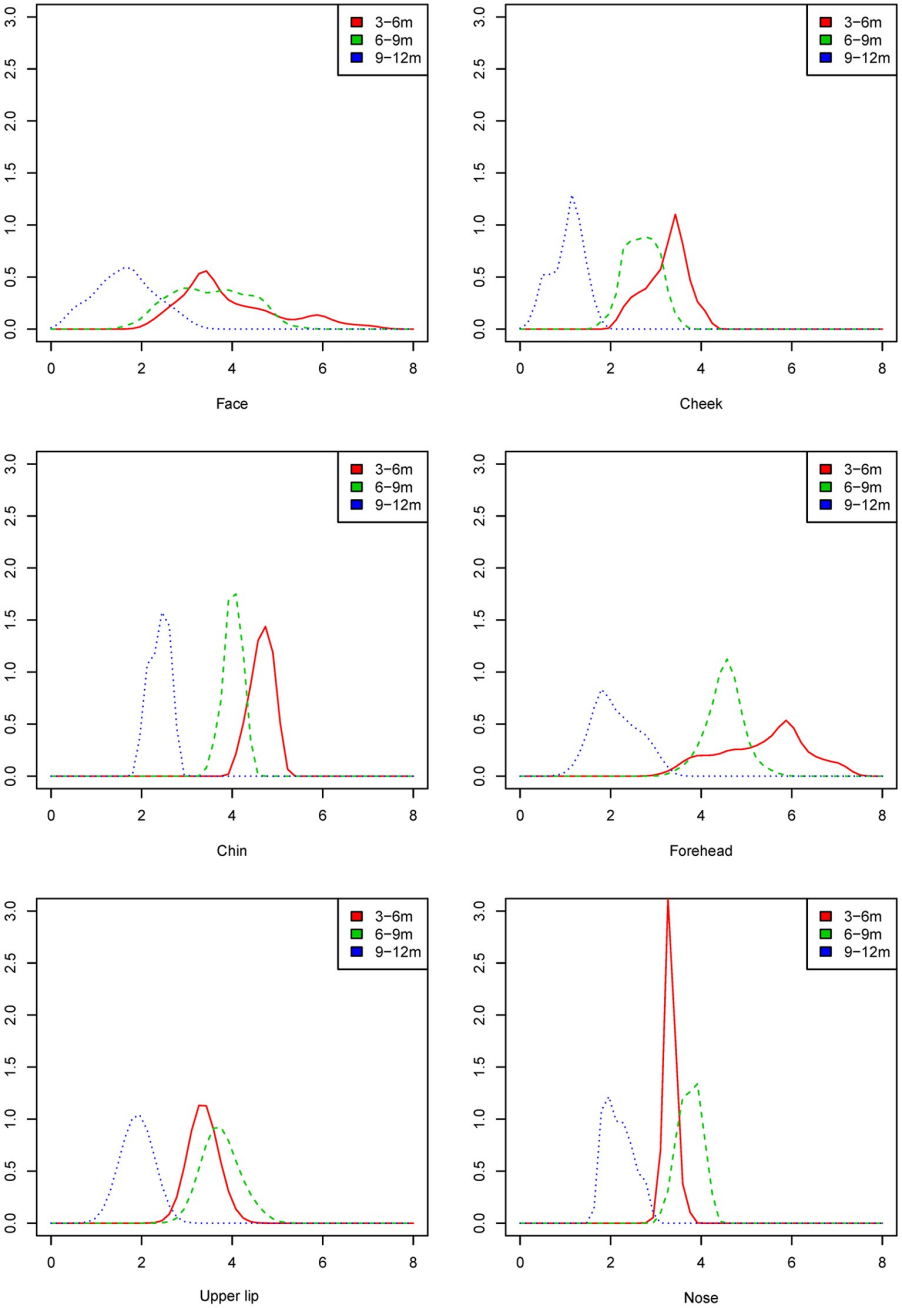
Density plots of growth of the total facial surface and facial regions at intervals 3–6, 6–9 and 9–12 months of age (*x*-axis growth in mm and *y*-axis in unit 1/mm).

Density plots are presented to describe the distribution of growth velocities within the total facial surface and the facial regions (Fig 5). For the total facial surface, there was a tendency of most of the facial surface growing 3.0–4.0 mm at intervals 3 to 6 and 6 to 9 months of age. At interval 9 to 12 months, most of the facial surface grew to 1.0–2.0 mm.

The density plots of the regions of the nose, upper lip, and chin show that these regions have the smallest distribution of growth velocities at all three intervals, indicated by the presence of a narrow and high peak. The density plots of the total facial surface and the forehead at intervals 3 to 6 and 6 to 9 months of age had the largest distribution of growth velocities demonstrated by the absence of a narrow and high peak.

## Discussion

This article describes a technique to develop normative average 3D faces of healthy infants at 3, 6, 9 and 12 months of age. Additionally, normative average 3D facial growth data from 3 to 12 months of age were obtained.

### Average faces

To date, there are no reference values for healthy babies for 3D craniofacial growth and development [23]. Therefore, it is unknown how many subjects are required for reliable development of an average face at ages 3, 6, 9 and 12 months. Some studies used 3D data in older age groups. Djordjevic et al. studied 17 male and 18 female healthy children, mean age 5.5 years, for a single average face [24]. Kau et al. studied 42 boys and 30 girls with normal facial features with a mean age of 11.8 years [25]. Bugaighis et al. studied 80 male and female control subjects with a mean age of 10.5 years [26]. From our longitudinal prospective 3D facial imaging database of 50 healthy control subjects, 16 to 28 high-quality 3D images were included per time interval. To obtain a sufficient sample size, we did not differentiate between boys and girls. This seems to be feasible since sexual dimorphism in the soft tissue of the face in babies has not been demonstrated in contrast to sexual dimorphism in adolescents and adults [26]. The only study in 3-month-old babies [27] reported differences in some facial dimensions but these differences were related to differences in weight between subjects rather than to sexual dimorphism. Only two studies have examined craniofacial sexual dimorphism in older children. Gaži-Čoklica et al. reported sexual dimorphism in the measurements of the cranial vault width and length, and facial height in children at 4.7 years of age [28]. Ferrario et al. demonstrated differences in soft-tissue facial dimensions between boys and girls as young as 6 years of age [29]. White et al. found a sex difference of 1–2 mm in larger facial measurements such as face height and ear-to-chin distance in infants 3 months of age, but this correlated with body measurements. There were no sex differences in the nose/upper lip width ratios or angular measurements, indicating there may be little sex difference in shape [30].

In a recent paper, a web-based repository (3D Facial Norms) was described that contains various craniofacial measurements derived from 3D facial surface images of over 2400 healthy participants from 3 to 40 years of age [31]. Measurements include standard anthropometric measurements obtained with a spreading caliper, inter-landmark distance calculated from 3D stereophotogrammetry, and 3D coordinate values (x, y, z) of soft tissue landmarks obtained from 3D stereophotogrammetry. The 3D Facial Norms database is an interesting initiative, and subsequent collection of 3D facial norms of children under the age of 3 years is highly desirable.

### 3D image processing

A uniform mesh pattern enables direct cross-sectional and longitudinal comparison of 3D facial images without the need for manual selection of landmarks on each individual 3D facial image, thus reducing operator error in the evaluation of growth. However, a selection of facial regions on the general face template is a manual task and thereby an operator error of unknown size is introduced. The variation in the amount of growth in the facial regions, however, is small compared to the mean growth. The standard deviations for the nose, upper lip, chin, forehead, and cheeks are 0.1–0.3 mm, 0.4–1.1 mm, 0.2 mm, 0.4–1.0 mm, and 0.3–0.5 mm, respectively. These values are small compared to the values of the mean growth. Therefore, mean growth is a reliable indicator of actual growth and the influence of an operator error due to manual selection of facial regions seems to be insignificant. Former studies generally used a best-fit-alignment with an algorithm that minimized the distance between 3D images [25, 26] or used a fixed landmark on the external facial surface such as the mid-endocanthion point [24]. This in turn directly influenced the amount of growth in the *z*-axis of the Cartesian coordinate system. The children’s reference frame, as used in the present study, registers 3D images on the average *z*-coordinate of the right and left preauricular point, which intends to resemble the traditional and biological registration on the cranial base. The children’s reference frame is comparable to registration on the cranial base. Comparison of the results of our study to the results of other studies is challenging due to differences in the measurement techniques. We recommend the use of the children’s reference frame for the cross-sectional and longitudinal evaluation of changes due to growth or treatment. This is more informative than simplifying facial dimensions into inter-landmark distances and angles. Registration on the children’s reference frame combines changes in both shape and size.

### Visual assessment of facial growth

The color-distance maps as presented are useful for a fast overview of the increments of facial growth in various regions of the face and a comparison of growth for different age intervals. The color-distance maps show relatively symmetric facial growth for the left and right sides as one would expect. This is a clinical confirmation of the reliability of the children’s reference frame [18]. Differences in growth between 6 and 9 months of age were noted at the periphery of the cheeks (Fig 4b). This was not found in the other age intervals. This may be due to a registration error or an actual facial asymmetry of one or more of the included subjects. We recommend that the data of the most lateral parts of the cheeks be interpreted with caution. Results for the nose, upper lip, and chin demonstrate well-defined symmetric growth at all intervals. In general, we found the most growth occurring at the youngest age intervals 3 to 6 and 6 to 9 months, and less growth from 9 to 12 months of age. The fastest growing region was found to be the forehead, representing the growth spurt of the brain [32]. The region of the chin grows fast as well, indicating the presence of some postnatal mandibular growth spurt.

### Quantification of growth of the face and facial regions

Mean growth of the full face was greatest in the 3- to 6-month age range, comparable to the growth at the 6- to 9-month age range. From 9 to 12 months, growth velocity decreased by half. Since the total facial surface included all vertices of the entire 3D facial image, it was indeed expected that the standard deviation would be higher than in the separate regions of the face. Mean facial growth from 3 to 12 months of age was found to be approximately 9 mm. Direct comparison with results of anthropometric studies was difficult because of age differences of the samples as well as the use of landmarks in conventional anthropometry [11] versus the use of facial regions in our study.

Mean growths of the nose and upper lip demonstrated a similar growth pattern with the largest growth at 3 to 6 months of age, and smallest at 9 to 12 months of age. The standard deviations of growth in the region of the nose, upper lip, chin, and cheeks were significantly smaller than that of the mean growth of the total facial surface. These indicated less variability of growth velocity in these regions and a very accurate indication of actual growth expressed by the mean growth. Mean growth of the forehead was 7 mm from 6 to 12 months of age, representing the postnatal growth spurt of the brain [32]. Farkas found the growth of the sagittal length of the head to be approximately 7 mm at the same interval of 6 to 12 months of age [11], validating our growth model.

The shape of a density plot for the full face or a separate facial region would ideally be narrow and high. This would indicate that the growth of the face or a facial region is likely to be highly predictable and would have little variation and a small standard deviation. The chin, nose and upper lip are the facial regions with the smallest variation and, therefore, have the highest probability in identifying actual facial growth independent of an accurate landmark selection. This justifies the use of facial regions as done in this study.

In the near future, the proceeding collection of 3D facial images of subjects before and after treatment as well as longitudinal control samples in online databases such as FaceBase, might enable Big Data analysis to predict growth and treatment outcome more accurately than we are able to do now. Currently, studies including large samples have demonstrated the potential to estimate age from 3D facial images and synthesize facial growth in children [33].

### Limitations

Average faces and mean growth data from 3 to 12 months are only applicable to Caucasians and healthy infants. Further research is needed to identify 3D facial growth data from 1 to 6 years of age, to assess facial growth in non-Caucasian populations and in populations with craniofacial malformations. We were unable to collect 3D facial images of newborns immediately after birth as it is practically nearly impossible to take images at that age. Therefore, the mean growth data from 0 to 3 months are unavailable.

Selection of the facial regions is a manual process which possibly introduces a reproducibility error. To reduce the reproducibility error, selection of the regions should ideally be performed automatically.

## Conclusions

This study describes the development of average faces at 3, 6, 9, and 12 months of age in healthy infants and estimates facial growth during the first year of life using 3D-stereophotogrammetry. Normative data on the growth of the full face, nose, upper lip, chin, forehead and cheeks have been presented. These data could be used in future studies to identify the effectiveness of various treatment protocols for orofacial deformities at an early age.

## Supporting information

S1 File 180710_ResultsControls.xlsx. Distance kits for the total facial surface and facial regions at intervals 3 to 6, 6 to 9 and 9 to 12 months of age.
(XLSX)
